# The Ophthalmoscope

**Published:** 1854-09

**Authors:** 


The Ophthalmoscope.—*Sir:* On leaving England, the author of the following paper desired me to forward it to you, with a request for its publication. In complying with his wish, I think it only right to mention that, although Dr. Williams omits to notice the ophthalmoscope of Coccius, Dr. Anagnostakis himself has described not only that instrument (which certainly embodies the principle of his own), but likewise all others which have preceded it.

Retaining, as I do, a very strong opinion as to the mischief likely to result from the abuse of reflecting ophthalmoscopes, if indiscriminately resorted to by inexperienced persons, for investigating cases hastily classed as "incipient amaurosis." I have no doubt that a series of ophthalmoscopic examinations, made by accurate and prudent observers, would tend to elucidate many obscure points in the pathology of the eye.

A practical inconvenience I have met with, in using the reflecting mirror of Dr. Anagnostakis, is, that it occupies one hand of the observer; so that if, at the same time, he wishes to employ with the other hand a convex lens, to magnify the structures he is illuminating, his examination, unless an assistant be present, may sometimes be foiled through inability on the patient's part to raise the upper lid sufficiently to expose the pupil.

It has occurred to me, therefore, to fix into a spectacle-frame, to be worn by the observer, a pair of reflecting mirrors, differing from those employed by Coccius and Anagnostakis only in being of less diameter, and having a smaller central hole. By this contrivance both hands of the observer are left at liberty, and he is enabled, without assistance, to command the movements of the patient's lids with one hand, while with the other he uses the magnifying glass.

The focus of the mirrors should nearly correspond to the distance at which the observer's unassisted eye can ordinarily define minute objects. I think it better to place the lamp immediately behind the patient, rather than by his side, and at such a height that the rays of light just clear the top of his head. The observer, in making his examinations, will find it best to use only one of his eyes at a time; but two mirrors are desirable, as they balance each other in the frame. Where a less concentrated light is preferred, plane glasses, silvered at the back, may be used instead of concave mirrors; a small round portion of the silvering being removed from the centre for the observer to look through.

I am, &c.

JAMES DIXON.

---

THE OPHTHALMOSCOPE.—*The principles on which it is based; the manner of its application, and its practical advantages, with a report of some cases.*—By E. WILLIAMS, M. D., of Cincinnati.—Among the many modern discoveries which have given a new impulse to the progress of medical science, nothing has been more effectual than the discovery and perfecting of the methods of physical diagnosis. Enabled, by these valuable means, to determine, with precision, the seat and gravity of pathological changes, it is no longer necessary to resort to those vague and bewildering hypotheses under which, formerly, the physician took refuge whenever he encountered a deep seated and obscure disease. What astonishing advancements have been made in the differential diagnosis of diseases of the chest since that cavity has been rendered, as it were, transparent by auscultation and percussion! But it is in ophthalmology especially that physical exploration has very recently undergone the most striking and important developments. Notwithstanding the high degree of perfection to which this beautiful branch of surgery has been brought by the researches of modern observers, there still remained till lately one lamentable deficiency. Unable to examine directly the cavity of the globe of the eye, the surgeon was obliged to rely upon indirect symptoms for the diagnosis of many of the most serious affections of that organ. Thus, at an epoch when medicine and surgery everywhere diligently sought the lesions of structure that predominate in each disease, amaurosis, sthenic, asthenic, congestive, nervous, gouty, dartrous, etc., were still seen arrayed in the nosology of the ophthalmologist, terms by which the ingenious surgeon disguised his ignorance when the local lesion escaped his means of investigation.

The discovery, therefore, of a mode of examining directly the interior of the eye in the living subject is by far the most important improvement made in ophthalmology in modern times, and is destined to form an interesting epoch in the history of this science. The parts situated in the centre, and at the bottom of the eye, heretofore inaccessible to the view of the observer, may now, by the aid of the ophthalmoscope, be subjected to the most minute inspection, and their separate lesions recognised with the greatest precision.

The practical consequences, as regards diagnosis, prognosis and consequently, treatment, following the invention of an instrument whereby this obscure cavity may be lighted up and examined, are of the highest importance. When, in this manner, we have inspected, one by one, all the parts in the interior of the eye without finding a lesion sufficient to explain the derangement of vision, then, and not till then, are we justified in assuming a more deeply seated alteration, or in resorting, to the hypothesis of an amaurosis *sine materiâ.*

The introduction of the ophthalmoscope opens up a new field for investigation. Should I, by this little essay, contribute to direct the attention of my professional brethren in England and America to this newly acquired territory, and stimulate some of them to explore and develope its rich resources, my object will be attained.

Setting out with this view, I hope to be able to serve the interests of the profession by giving a concise idea of the principle upon which this new method of examination is based; of the construction of the ophthalmoscope; and of the practical results to be derived from it.

*Principle upon which the construction of the Ophthalmoscope is founded.*—The pigmentary layer of the choroid is not sufficient to account for the black appearance of the bottom of the eye as seen through the pupil. The pigment would, indeed, absorb all the rays of light that enter the eye, were there no tissues situated anteriorly to it, capable of reflecting a portion of the rays. The vessels of the retina, and the optic nerve at its point of entrance, must certainly have this power. Besides, this, however transparent the retina may be supposed to be during life, a membrane so brilliant must necessarily reflect a large number of the luminous rays that fall upon it.

*Why, then, does the pupil ordinarily appear black?*—It is only in the refracting properties of the eye that the explanation of this phenomenon must be sought. Let us suppose that the eye to be examined looks at a luminous point. The rays projected into the eye from this point, being refracted, will be concentrated at a point on the retina and reflected in their turn by this membrane, they will again issue from the organ. But, since they must pass through the same media they had traversed on entering, they will undergo the same refraction, and be consequently collected at the point from which they first departed, in order to form there the retinal image.

From this it follows, that we could see the retina of an individual only when he looked attentively at our own eye, which, in that case, would be the luminous point. But it is clear, that the quantity of light which our own eye can project is too feeble to illuminate the bottom of the eye explored; and, in attempting to look into the interior of that eye by the aid of ordinary daylight, we only intercept with our head the rays which should illuminate the cavity. Placed, in this manner, in the shade, the pupil will naturally appear black.

After these considerations, it is easy to comprehend the object to be kept in view in using the ophthalmoscope. It is to throw into the eye, indirectly, a large quantity of light, in such a way that the observer, without intercepting the rays which enter, may receive directly into his own eye those reflected from the retina of the explored organ. With this view, any mirror may be employed, provided it be held obliquely with respect to the eye examined, and have a transparent spot, or a round hole, through which the observer may look.

*Construction of the Instrument.*—To Helmholtz, Professor of Physiology at Königsberg, is due the invention of the first ophthalmoscope. The limits of my paper forbid a detailed description of his instrument. It is only necessary to add, that this ophthalmoscope, with all the modifications to which it has been subjected, has but very imperfectly fulfilled the desired end; since not only the illumination which it affords is insufficient, but its complexity also renders it extremely difficult of application. My present object is, simply to describe the instrument of Dr. Anagnostakis, of which an account will be found in his original and admirable monograph above cited. Combining, as it does, the entire advantages of all the complicated and expensive ophthalmoscopes heretofore invented with the utmost degree of simplicity, facility of application, and portability, it will, without doubt, wholly supersede them. It is now exclusively used in France and Belgium, and the results of its application have been so satisfactory as to leave little else to be desired.

The instrument is composed of a small, round, concave mirror, about two inches in diameter, with a focal distance of four and a half inches; the silvered surface being accurately adapted to a plate of blackened copper. The centre of this little mirror is perforated by a small hole about three lines in diameter. Around the border of the glass and plate thus adapted is placed a brass rim, to which is attached a small handle of wood or ivory.

*Method of using the Ophthalmoscope.*—The pupil of the eye to be examined, if not already very large and fixed, should be previously dilated with atropine or belladonna. A dark room is selected; on a level with the patient's eye a bright lamp is placed upon a table as near as possible to the patient, who is seated beside it. The observer is seated upon a chair close in front of him, so that their eyes shall be upon the same level, and holds the mirror with the reflecting surface directed in such a manner that the rays reflected from the lamp are thrown upon the eye to be examined. Then, applying his eye against the central hole, the observer looks through it into the bottom of the eye thus illuminated; and, still holding the instrument constantly and steadily against his eye, he approaches towards or recedes from the patient till the reflection becomes small, brilliant, and oblong in form, and the tissues at the bottom of the globe are recognized. If it is desirable to magnify the image of these parts, or if, in consequence of the extreme presbyopia of the patient, the observer is obliged to place himself at a great distance in order to see the deep tissues of the eye, a convex lens of suitable magnifying power must be used. Accordingly as the refracting power of the eye examined is greater or less, so must the eye of the surgeon be brought nearer to or removed further from the patient, in order to have a clear image. But to secure, at the same time, a sufficient illumination, the focus of conjunction of the mirror must fall upon the cornea of the eye observed. Since, however this focus, in a concave mirror, varies with the distance of the light from the mirror, by altering that distance, the focus may be lengthened or shortened at pleasure. Thus the instrument may be adapted to all eyes indiscriminately, whether myopic or presbyopic.

These are the general rules to be observed, and a little practice will give the surgeon that facility in the use of the instrument necessary to accurate observation.

A few words with regard to the appearances presented by the healthy eye are necessary to enable the reader to appreciate the pathological changes which the ophthalmoscope reveals.

On first looking, as above directed, into an eye free from disease, the pupil is seen of a reddish color, but none of the deeper structures are distinguished. As the parts at the bottom of the eye are necessarily seen through a refracting medium, which varies in degree in different individuals, it is evident that the surgeon must approach towards or recede from the eye inspected, till the proper focus is found; he will then see red vessels traversing the bottom of the organ. When these blood vessels are distinctly recognised, it is certain that the proper focal distance is attained, and the retina is in view.

This point, once gained, should be steadily preserved, while the patient is directed to turn his eye-ball in different directions; thus all the parts of the retina are successively brought into the field of vision. When the globe is turned upwards and inwards, a spot usually circular, is seen, much whiter than the surrounding parts, with a small umbilicated indentation in the centre, through which pass four blood vessels (two directed upwards, and two downwards), which branch off to the different parts of the membrane. But very frequently these principal trunks divide before entering the globe, and then a larger number of vessels are seen coming in at the white spot formed by the entrance of the optic nerve. Except this patch, which is quite white, the rest of the retina presents a reddish color, which becomes more decided towards the periphery.

I must now speak of the pathological changes which have been already revealed by the aid of the ophthalmoscope. I shall adopt the natural order of noticing, first, the lesions that have their seat in the crystalline lens and viterous humor; second, those confined to the essential organ of vision, the retina.

I imagine that no opacity of the lens, however slight, can escape detection by the use of this instrument. Certain it is that opacities which even materially interfered with vision, but which escaped the scrutiny of the most experienced eye, have been readily detected by a resort to the ophthalmoscope. Some very eminent ocultists have reported cases of this kind. I have seen, at the clinic of M. Desmarres, beautiful opaque streaks in two lenses, which had eluded all other means of examination. When seen by the aid of this instrument, opacities of the crystalline appear as greyish-brown patches or streaks, of various sizes and shapes, upon a reddish back-ground, afforded by the illuminated retina. The importance, as to treatment and prognosis, of distinguishing the very commencement of cataract from impairment of vision produced by other more serious lesions of the vetreous or of the retina itself, is too obvious to need illustration. Who that has had even a limited experience in diseases of the eye has not seen patients with incipient cataract, tortured for months or years with leeches, cupping, blistering and a thousand pernicious drugs, under the supposition of a commencing amaurosis? And who has not been consulted by patients sent to be operated upon for cataract, in whom the lens was perfectly transparent?

The most common alteration of the vitreous humor is the appearance in its substance of deep brown or greyish corpuscles, in greater or less abundance. These bodies are of very various sizes and shapes. Sometimes we see only two or three large, irregular flakes, either floating separately in the liquid vitreous humor, or united to each other by a number of small filaments. At other times an immense number of smaller corpuscles, some angular, others in the form of shreds, and others, again, resembling pieces of membrane with ragged edges. When agitated by the sudden movements of the globe, these floating bodies are seen bounding and darting about in the interior of the eye, and gradually subsiding towards the inferior part when the organ is at rest.

Whether these substances are inflammatory exudations from the ciliary body, retina, or choroid, that have become detached; whether they are the remains of blood effused into the cavity of the eye; or, as is more probable, attributable sometimes to one and sometimes to the other of these sources, is not yet settled. Future observations can alone decide that question. Since, in all cases where these corpuscles exist, the vitreous humor is disorganised and liquid, I have thought that some of them might be opaque and thickened portions or shreds of hyaloid membrane.

Doubtless many cases of muscæ volitantes are produced by these little bodies floating before the retina. As, however, they are not unfrequently seen in patients who are not at all troubled with muscæ, and are absent in others where this phenomenon is complained of, they cannot be considered as its sole cause. If small, and occupying the anterior segment of the vitreous body, they may throw no shade upon the retina, and hence not interfere in the slightest degree with the vision. But when of large size, and situated in the posterior segment, near the retina, they must necessarily derange the sight by mechanically intercepting the rays of light.

As examples are always more interesting and instructive than general descriptions, I will give a short account of some cases which I have had occasion, within the last few weeks, to observe at the London Ophthalmic Hospital, in the presence of Mr. Dixon and Mr. Bowman, who have themselves seen some of the alterations which it is my object now to describe. It is with great pleasure that I take this opportunity of thanking the eminent surgeons of this Institution for the kind permission they have granted me of attending their extensive and interesting practice; and especially of continuing those observations with the ophthalmoscope which I had commenced at Paris with Dr. Anagnostakis, the ingenious inventor of the ophthalmoscope which I use. The few cases inserted in this paper were also examined by the latter gentleman, and his experience in the use of the instrument is an additional guarantee for the accuracy of these observations.

---

Case 1. John C., aged 38, a farrier, states that fourteen years ago he received an incised wound of the lids of the right eye, but the globe was not touched, nor the sight injured. About the 1st of February last, a hammer-head, flying from the handle, struck him in the eye, rendering him for a few seconds insensible. On the retun of consciousness he could not see with this eye. Two days after the accident, he applied at St. Thomas's Hospital, and was told his eye was destroyed. Leeches, fomentations, etc., were resorted to for the relief of suffering. Some time afterwards he came to the Royal London Ophthalmic Hospital to consult Mr. Poland, and has been since under his care.

*Present Condition.*—The patient still suffers occasional neuralgic pains in the eye and head. With this eye he distinguishes large objects, but very indistinctly; says, however, that his sight is slightly improving. The iris is separated from its ciliary attachment at the upper and external part, in more than a third of its circumference, and also at the lower and outer part to a somewhat less extent. Thus detached, this membrane is stretched across the aqueous chamber in the form of a rather narrow band, above and below which the bottom of the eye is seen, presenting a black appearance as through the ordinary pupil. With the naked eye, nothing else can be observed; but with the ophthalmoscope a number of fine filaments, as of lymph, are seen extending from the inferior edge of this band to the original attachment of the iris. To the cilary body above, where the iris has been detached, adhere by one extremity a number of shreds that float in the aqueous humor. In the vitreous humor, many brownish flakes are seen floating freely about; they subside slowly to the inferior part of the cavity when the eye is quiet, and, when it moves, mount up into the vitreous again. The retina, as indistinctly seen through the turbid refracting media, has its normal appearances. In this case the impairment of vision is evidently not dependent upon a lesion of the retina, but upon the mechanical impediment to the passage of light to that membrane by the separation of the iris and the foreign bodies in the vitreous humor. These corpuscles are doubtless flakes of effused lymph, or clots of fibrin from the effusion of blood at the time of the accident. The extravasation of blood into the vitreous humor, except as a consequence of mechanical violence is very rare. In a case, however, which I examined some time since with Mr. Bowman, there were seen, by the aid of the mirror, beautiful clots of blood floating in the substance of this humor; but while we were examining other patients the man went away, and I have not since been able to see him, so as to procure the history of his affection.

As alterations of the vitreous humor are generally accompanied by lesions much more serious of the retina, I will give under another head some additional examples of synchysis with floating corpuscles.

*Lesions of the Retina.*—By far the most interesting pathological changes that occur in the eye are those involving the retina. The anatomical lesions that affect either directly or indirectly this delicate membrane are somewhat numerous; but I shall notice a few only of the most prominent changes, and such as are very readily detected by the mirror.

*Dropsy of the Retina.*—By this is meant an effusion of a serous fluid beneath the retina, which detaches it from the choroid, and thus entirely annihilates its functions.

---

Case 2.—Thomas W., aged 38. As this patient is completely deaf, but little definite information could be collected as to his previous history. It seems that about sixteen years ago, after falling through the ice, he began to suffer pain in the right eye, with headache, giddiness, etc., and his sight failed so rapidly that in a few weeks he could distinguish no objects. Since that time his eye has remained in about the same condition. He still has occasional pains in the globe, and headache.

*Present Condition.*—The left iris is somewhat discolored, the pupil preternaturally dilated and sluggish; sub-conjunctival vessels enlarged, tortuous, and of a bluish color. This eye is very myopic, and the sight much impaired, but at a distance of about two inches he reads clear type.

The pupil of the right eye is largely dilated and fixed. There is varicosity of the sub-conjunctival veins, and a leaden hue of the sclerotic; lens transparent. With the naked eye nothing else is seen. No perception of light.

With the ophthalmoscope the vitreous is seen to be slightly opaque, and containing a considerable number of brownish bodies resembling flakes of lymph, which are constantly agitated by the movements of the globe. The entire retina, separated from the choroid, is pushed forward by a clear liquid in the form of a pouch, which surrounds the entrance of the optic nerve, not unlike the air cushions used for sitting on in travelling. The entrance of this nerve is seen as a yellowish-white, circular spot, through which pass three blood-vessels, which mount up over the pouch formed by the retina, and, dividing into small branches, spread over its surface. This liquid pouch is thrown into beautiful undulations by the movements of the globe, and the vessels running over its surface alternately bend upon themselves, and elongate with the folding and unfolding of the waving retina.

There is here a large effusion of serum between the retina and the choroid, which has separated all their attachments, and entirely destroyed the power of perceiving even the strongest light.

---

Case 3.—James F., aged 45, of robust constitution, enjoyed good sight till about four months ago, when, in running violently, he fell and received a severe shock. About three weeks afterwards accidentally covering the right eye with his hand, he perceived to his astonishment, that he could not see with the left. He has never experienced the slightest pain or uneasiness in this eye. Of objects placed before or to the right side of him he has not the faintest perception; but he recognizes very imperfectly large bodies situated towards his left side. The pupil is more dilated than the other, and immovable.

Examined with the mirror, the refracting media appear perfectly transparent. The retina presents no abnormal vascularity, but it is easy to recognise that this membrane, to a large extent, and all around the entrance of the optic nerve is elevated by a liquid, and it has a trembling movement during the oscillations of the eye. What is curious in this case is, that during these movements we do not see those deep folds which the membrane ordinarily forms in dropsy of the retina. Here the folds are superficial, and the undulations quite limited. These phenomena, taken in connection with the pearly color which the elevated membrane presents, can only be explained by supposing that the retina is raised up by a turbid liquid, as we often observe in pericarditis and other serous inflammations.

---

Case 4.—John R., aged 48, has been from childhood subject to blepharitis, but enjoyed good sight. About fourteen years ago he received an injury of the left eye from the explosion of a soda-water bottle. For some months afterwards he had inflammation and pain in the eye; these at length passed off, and left his vision unimpaired. Nearly nine years ago, he received a blow upon the same eye with a fist, and instantly ceased to see. This injury was followed, during several months, by severe pain, which finally ceased. For several years he has been an occasional attendant at the Hospital for the blepharitis to which he is subject.

*Present Condition.*—The form and consistence of the globe are natural; conjunctiva, sclerotic, and cornea, healthy. The patient has not any perception of light, except when a bright lamp is held towards his right side; the iris, at the internal part, for nearly two-thirds of its circumference, is separated from its ciliary attachment. With the naked eye, in a strong light, when sudden movements are communicated to the globe, vague and undefined undulations are seen at the bottom of the eye.

*Examination with the Ophthalmoscope.*—A great number of corpuscles are seen floating in the substance of the vitreous humor. The crystalline lens, as proved by the catoptric test, has entirely disappeared. Nearly the internal third of the retina is detached from the choroid, and pushed forward into the vitreous humor, in the form of a vesicle, filled with transparent liquid. When the globe oscillates, this vesicle is thrown into waves, which disappear when it is still. Two blood vessels pass over, and ramify minutely upon the surface of this pouch, and, during its undulations, alternately become curved and straight, not unlike the long grass at the bottom of a clear running stream. The entrance of the optic nerve presents its usual appearance, but the part of the retina not detached from the choroid has a slightly opaque appearance, as if its meshes were infiltrated with a milky fluid.

Of the three well-marked cases of dropsy of the retina above described, there was only one in which the slightest appearance of the disease could be detected with the unaided eye. In the last mentioned patient, the iris was detached to so great an extent as to allow the entrance of a great quantity of light, and even then only a faint glimpse of the waves of the detached retina could be perceived by the naked eye. With the mirror, nothing was easier than to see at once the character and extent of the lesion in all the cases. No magnifying glass was used. It would seem that th is one of the commonest, and certainly most serious affections of the retina, as many cases of it have been seen, and no cure has as yet been published. It is not probable that the retina, once detached from its connexions with the choroid, can ever again resume its functions.

---

CASE 5.—*Varicosity of the Vessels of the Retina, with Ecchymosis.*—Alfred C., aged 12, of lymphatic temperament, has had very imperfect vision since birth, and has been taught to read raised letters by the touch. He is so extremely myopic that he is obliged to place objects at a distance of one inch only from his eye in order to see them. At that distance, he recognizes very large letters. There is a constant oscillatory movement of both globes. The irides are of a natural color; the pupils are but little influenced by the stimulus of light; the left pupil is larger than the right. The pupils having been dilated with atropine, the eyes were examined with the ophthalmoscope. The refracting media are perfectly transparent. One sees the retina at the first glance, from a very short distance,—a phenomenon which is only explained by the extreme myopia or prolongation of the antero-posterior axis of the eye. The vessels of both retina are so much developed as to appear varicose, and here and there are seen patches of ecchymosis, which are especially numerous and large in the right. At the inferior part of the left retina, places are observed where that membrane seems atrophied and thinned, and permits the tissue of the choroid to appear through it. The vessels in these places are evidently elevated above the level of the membrane. I neglected to state that this boy cannot distinguish colors, even if he has any idea at all of them. His sight is not improved by concave glasses, but rather made worse; but with a convex lens, he sees a little better. If this imperfect vision depended, as it does in some cases, upon the extreme myopia alone, the sight would be improved by concave, and made worse by convex glasses; the reverse of which is here the case. There must then be a congenital defect of the retina, although no very striking deviation from the natural appearance is seen by the aid of the ophthalmoscope.

Effusions of blood in the substance of the retina, as in this case, are very common, particularly in persons predisposed to congestion of the choroid.

The extent of these ecchymoses varies from that of small specks to patches involving nearly the whole of the retina. Extravasation of blood into the substance or beneath the retina, is, doubtless, the cause of many cases of sudden amaurosis.

---

CASE 6.—*Brown spots upon the Retina.*—William A., aged 52, about two years ago, was seized with partial loss of sensibility in the inferior extremities, which still persists. He has occasional cramps in the legs, and some difficulty in retaining his urine and fœces. Some twelve months since he remarked that his range of vision was diminishing—that he could only see well immediately in front of him. This narrowing of the field of vision has increased till now.

*Present Condition.*—Range of vision in both eyes very limited. He cannot see two points that are separated more than an inch and a half from each other. The third pair of nerves partially paralyzed. Iris natural and active in both eyes.

With the mirror, the refracting media are found transparent. Both retina are spotted with deep-brown patches, which are most numerous towards the periphery; but there are some immediately around the entrance of the optic nerves. What these brown spots are is not determined. They may be the remains of old ecchymoses, or else accumulations of pigment, such as are sometimes seen along the course of the vessels of the subconjunctival cellular tissue. These brown specks are very frequently observed in cases of impaired vision from congestion of the choroid; and, being frequently found intermingled with patches of recent ecchymosis, it is not improbable that they are the remains of old extravasations of blood.

The derangement of vision in this patient is much greater than can be explained by the alteration observed in the retina, and hence must be attributed to a lesion more deeply seated, the existence of which is rendered certain by the general symptoms.

---

Case 7.—*Greyish-white patches upon the Retina.*—Mary J., aged 67. Has always been myopic. She says her right eye has been weaker than the left since childhood. In 1841, she fell down in a fit, probably epileptic, and struck the right eye upon the corner of a box. About twelve months afterwards she had another similar fit. She has a cataract in the left eye, which dates from 1842, and which now seems to be undergoing gradual absorption, and the sight of the eye is slightly improving. As to the right eye, she is not aware that she has ever had cataract in it, but says the sight of it has been much impaired for many years. The lens has been dislocated, either spontaneously or by the force of the fall she has had, and is now seen, much reduced in size, lying in the inferior part of the vitreous humor, behind the iris, which possesses its natural color. The pupil is languid in its movements. With this eye she sees to find her way about the streets, and can discern large letters. There is dissolution of the vitreous humor as is proved by the trembling of the iris during the movements of the eye.

With the ophthalmoscope is seen (as also with the naked eye)

the nucleus of the right lens lying at the bottom of the globe. In the liquid vitreous humor are observed a number of brownish corpuscles of various forms and sizes, which are constantly agitated by the oscillations of the globe.

But the most interesting alterations are in the retina. It is seen at the first glance, and from a very short distance, without the aid of a convex glass, traversed by a great number of extremely developed blood vessels. The white patch formed by the entrance of the optic nerve is very small, but still quite distinct. A little above, and externally to this point is recognised an irregular patch, with perfectly circumscribed border, about two lines in its longest diameter, and of a pearly tint. This patch is slightly elevated above the level of the rest of the retina, and is streaked with a large number of varicose vessels, among which are seen reddish-brown specs, apparently caused by extravasated blood. Smaller patches, pearly white, but offering, in other respects, exactly the same characters, are seen here and there in other parts of the retina. What the nature of this lesion is, it is impossible, in the present state of science, to say. It is certain that the retina at these points has lost its natural transparency; but, whether that depends upon a partially organized exudation beneath the retina, upon a degeneration of that membrane itself, or upon earthy deposits, we have as yet no means of determining.—*Medical Times and Gazette*, *July* 8, 1854.

